# Linking park environmental characteristics to child health outcomes: towards an evidence-based child-friendly design framework

**DOI:** 10.3389/fpubh.2026.1795835

**Published:** 2026-05-15

**Authors:** Lin Zhu, Li Wu, Xiran Xu, Xiangwei Hu, Yiqiu Su

**Affiliations:** 1 College of Architecture and Urban Planning, Hunan City University, Yiyang, China; 2 Key Laboratory of Urban Planning Information Technology of Hunan Province Universities, Yiyang, China; 3 International One Health Institute, Wenzhou-Kean University, Wenzhou, China; 4 College of Landscape Architecture and Art, Henan Agricultural University, Zhengzhou, China; 5 Department of Landscape Architecture, Faculty of Design and Architecture, Universiti Putra Malaysia, Serdang, Selangor, Malaysia

**Keywords:** child-friendly city, children’s health, health and well-being, park environment, spatial optimization

## Abstract

With the deepening of the Child-Friendly City concept in global urban governance, understanding how park environments systematically promote children’s physical and mental health has become a critical issue in both research and practice. Taking the urban park system of Dazhou City, Sichuan Province, China, as an empirical case, this study constructed a comprehensive evaluation framework encompassing six dimensions—safety, accessibility, comfort, playability, naturalness, and inclusiveness—to examine the relationships between environmental characteristics and children’s health outcomes. The results indicate that 14 environmental indicators significantly affect children’s physical health (*β* = 0.105–0.227), primarily through pathways related to risk reduction and physical activity promotion, among which noise control, facility diversity, and barrier-free design show the most prominent effects; In contrast, 9 indicators significantly enhance children’s mental health (*β* = 0.139–0.380), mainly via mechanisms associated with cognitive restoration and emotional regulation, with interactive engagement, wildlife habitat, and seasonal landscape exerting the greatest influence. These findings reveal a differentiated mechanism in which physical health is driven by stress mitigation and activity support, whereas mental health relies more on restorative experiences and human–nature interactions. Based on these empirical findings, this study further proposes cultural integration, and micro-environmental renovation as optimization strategies, providing practical and operable guidance for developing child-friendly parks in high-density urban contexts.

## Graphical abstract

**Figure:**
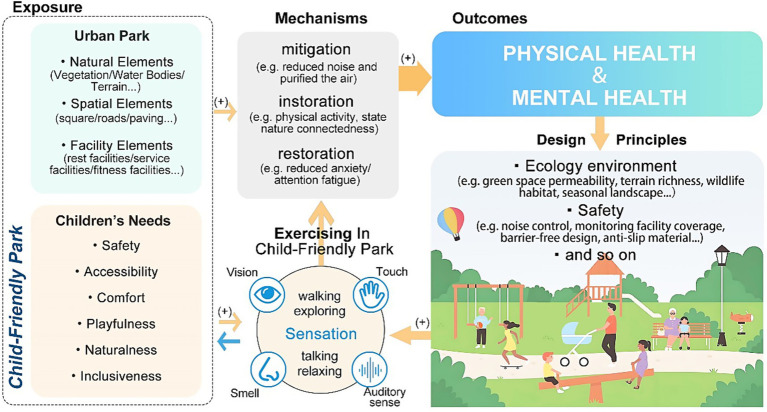
The diagram synthesizes the six core dimensions (safety, accessibility, comfort, playfulness, naturalness, and inclusiveness) and their hypothesized pathways to physical and mental health outcomes.

## 1 Introduction

As scarce yet highly accessible green spaces within high-density urban areas, parks play an irreplaceable role in promoting the physical and mental health of children. A substantial body of research has demonstrated that contact with natural environments can significantly improve children’s attention restoration and emotional regulation, enhance social interaction, and effectively reduce health risks, such as obesity (1, 2). This scientific consensus is consistent with the Child-Friendly City (CFC) concept advocated by UNICEF (2021), which emphasizes the integration of children’s rights to survival, development, and participation into urban governance frameworks, thereby safeguarding children’s holistic well-being via safe, inclusive, and nature-oriented spatial design.

Despite the growing body of research, significant limitations persist, constraining its applicability within high-density urban contexts. Theoretically, current studies on green spaces for children’s health often adopt a fragmented perspective, focusing on single environmental elements such as total green space area, vegetation coverage, or specific types of facilities. They lack a systematic deconstruction of how multiple dimensions, such as safety, naturalness, playability, and inclusivity, interact synergistically to shape children’s health outcomes. Empirically, existing evidence is primarily derived from low-density Western contexts, leaving the spatial-behavioral mechanisms in high-density, culturally distinct Asian cities underexplored. Consequently, how to achieve coordinated responses under the multiple challenges of limited land and diverse cultural needs remains a critical gap in current scientific understanding and design practice.

To address these gaps, this study selects Dazhou City in Sichuan Province, China, as an empirical case, a representative area advancing the “Park City” development concept. Theoretically, this study constructs a child health-oriented urban park environment assessment system by integrating multi-dimensional indicators with a health mechanism model. Practically, it investigates how parks systematically shape children’s health through their spatial characteristics and distribution patterns in high-density urban environments, thereby providing more robust evidence to support the construction of child-friendly cities. Based on this, the study proposes three core research questions:

- Which specific environmental characteristics of urban parks have critical impacts on children’s physical and mental health?
- Through what “space-behavior” pathways do these characteristics influence children’s health?
- How can these empirical evidences be translated into effective strategies that guide the planning and design of child-friendly parks?

### 1.1 The scientific link between children’s health and nature contact

Urban parks serve as crucial everyday activity spaces for children, and their environmental characteristics have become a core focus of interdisciplinary inquiry in public health, urban planning, and environmental behavior studies. Extensive empirical studies have shown that nature exposure not only directly improves children’s physical health but also exerts indirect impacts via diverse mental and behavioral pathways.

#### 1.1.1 Subjective measurement methods

Subjective measurement studies rely on self-reports as well as reports from parents or teachers, and hold unique value in assessing internalizing symptoms, quality of life, and social–emotional functioning. Regarding physical health, studies utilized the International Study of Asthma and Allergies in Childhood (ISAAC) questionnaire and found that dust, animal contact, and various agricultural activity patterns in farm environments act as either risk or protective factors for childhood asthma and respiratory symptoms (3). Kokkonen found through parental reports that frequent nature exposure is associated with longer nighttime sleep duration in children, yet shows no significant correlation with obesity (4).

In mental health, multiple studies employing the Strengths and Difficulties Questionnaire (SDQ) and parental reports have consistently confirmed that insufficient residential greening and less time spent playing in parks are significantly associated with poorer mental health in children (5, 6). Moreover, allocating approximately 21 to 40% of residential land to green space may represent the optimal range for promoting children’s well-being (7). The Pediatric Quality of Life Inventory (PedsQL) have been widely applied to evaluate the effects of nature-based interventions, demonstrating that environmental education, horticultural programs and other initiatives can improve health-related quality of life in children with dermatitis, as well as low-income and ethnic minority adolescents (8–11). Other subjective measurement tools, including the Self-Rated Mental Health Scale (SRHMS), the Kessler Psychological Distress Scale (K6), and the Patient-Reported Outcomes Measurement Information System (PROMIS), have also been utilized to confirm a significant negative correlation between exposure to the natural environment and symptoms of depression, anxiety, and insomnia in children (12, 13).

Furthermore, studies using tools such as the Mindful Attention Awareness Scale (MAAS) and the Nature Relatedness Scale (NR-6) have shown that nature exposure is closely associated with higher well-being, better quality of life, and lower stress levels in children (14–16). Participation in horticultural activities is linked to reduced depressive symptoms, improved emotional health, and enhanced family bonding in adolescents (17), though some studies have observed inconsistent or weak associations (18, 19). In mixed-methods research, the combination of Geographic Information System (GIS) and parental reports has revealed that low greening is associated with poorer mental health in children (5); the d2 Attention Test and well-being questionnaires found no immediate positive effect of green spaces on health (20); and the combination of the Normalized Difference Vegetation Index (NDVI) and the SDQ demonstrated that both green space exposure and moderate-to-vigorous physical activity are associated with higher emotional health in children (21).

#### 1.1.2 Objective measurement methods

Objective measurement studies adopt quantifiable and standardized tools, including GIS spatial analysis, direct physical measurements, standardized cognitive tests, and administrative health records. In spatial exposure assessment, numerous studies using GIS and the NDVI have confirmed that residential proximity to green spaces is associated with higher intelligence quotient (IQ) in children (22), lower risk of depression (23), lower rates of attention deficit hyperactivity disorder (ADHD) medication use (24), decreased hyperactive behaviors and improved visual memory (25), and enhanced overall cognitive ability (26). Green space accessibility is also closely linked to reduced risk of childhood overweight and obesity (27) and improved lung function (28, 29) further revealed that the beneficial association between green space exposure and cognitive development is partially mediated by a reduction in air pollution.

In physical and cognitive measurements, attention tests are widely used to assess the immediate effects of nature exposure. Multiple studies have confirmed that 20 to 30 min of nature exposure is sufficient to significantly improve children’s attention performance (30–33), and nature exposure can markedly alleviate symptoms in children with ADHD (34). Regarding cardiometabolic health, direct measurements of blood pressure, blood lipids, and blood glucose have confirmed that green space exposure can improve adolescent cardiovascular health (35), while after-school programs can promote cardiovascular health in children with intellectual disabilities (36). Neurophysiological research using electroencephalography (EEG) has demonstrated that creative activities in forests can alleviate stress and enhance self-esteem (37).

### 1.2 Theoretical framework of child-friendly space design

In the in-depth interpretation of the relationship between nature and children’s health, multiple theoretical perspectives have formed a complementary system. The Biophilia Hypothesis posits that humans have an innate dependence on and emotional connection with nature, and natural environments can elicit positive physical and mental responses (38). The Attention Restoration Theory (ART) and Stress Reduction Theory (SRT) constitute two primary theoretical frameworks for explaining the mental mechanisms underlying this relationship. ART posits that landscape elements in natural environments characterized by “soft fascination” can facilitate the restoration of attention resources depleted by daily tasks, thereby enhancing concentration and cognitive functioning (39). SRT further elaborates that exposure to nature can lower cortisol levels and sympathetic nervous tension, alleviating depressive and anxiety symptoms in children (40). Meanwhile, Affordance Theory has been introduced into child-friendly space research in recent years, which emphasizes the behavioral possibilities provided by spatial environments for children, such as gentle slopes afford climbing (41).

Driven by theoretical and empirical research, design practices of child-friendly spaces have evolved from conceptual advocacy to concrete spatial interventions. Early practices, such as Delft’s “Child Safety Plan” in the Netherlands, laid the foundational groundwork for safety, accessibility, and comfort by constructing safe commuting routes linking schools, homes, and parks (42). Chinese scholars have developed multidimensional indicat or systems based on different study objects and site types. For instance, children’s hierarchical needs spanning protection, accessibility, comfort, and enjoyment (43). Three dimensions of culture, order, and interest proposed for historical districts (44). Additionally, other studies have extracted key environmental factors such as safety, friendliness and health through the analysis of spatial form and behavioral satisfaction (45, 46).

In recent years, research has gradually shifted to exploring the dynamic coupling mechanism among space, behavior and health, driving the evolution of design frameworks toward an evidence-based and refined direction. For example, studies in Dortmund, Germany, have further emphasized the critical role of facility diversity in enhancing children’s physical activity and social competence (47). Research in North America has focused on the configuration of community green spaces, examining the combined impacts of vegetation coverage, path safety, and activity area design on children’s outdoor activity duration. Moreover, Numerous empirical studies have demonstrated significant positive correlations between community greening levels, the scale and functional diversity of public activity spaces, and children’s outdoor activity levels (46, 48). Factors such as commuting distance between home and school, accessibility to sports venues, and landscape quality directly impact adolescents’ daily physical activity (49, 50). The continuity of pedestrian systems, the connectivity of transportation networks, and the quality of sports facilities positively contribute to children’s physical development, while high floor area ratios may exert inhibitory effects (51, 52). Spatial environmental quality and accessibility to public service facilities exert measurable, evidence-based impacts on children’s health (53, 54).

Overall, relevant theoretical and empirical studies have jointly revealed the core mechanism by which park environments promote children’s health: urban parks respond to children’s multi-level needs in terms of safety, accessibility, comfort, playfulness, naturalness and inclusiveness through the optimization of natural, spatial and facility elements, and ultimately exert a positive promoting effect on children’s physical and mental health through multiple pathways of mitigation, restoration and intervention (Graphical abstract).

## 2 Research methods

### 2.1 Research object

**Figure 1:**
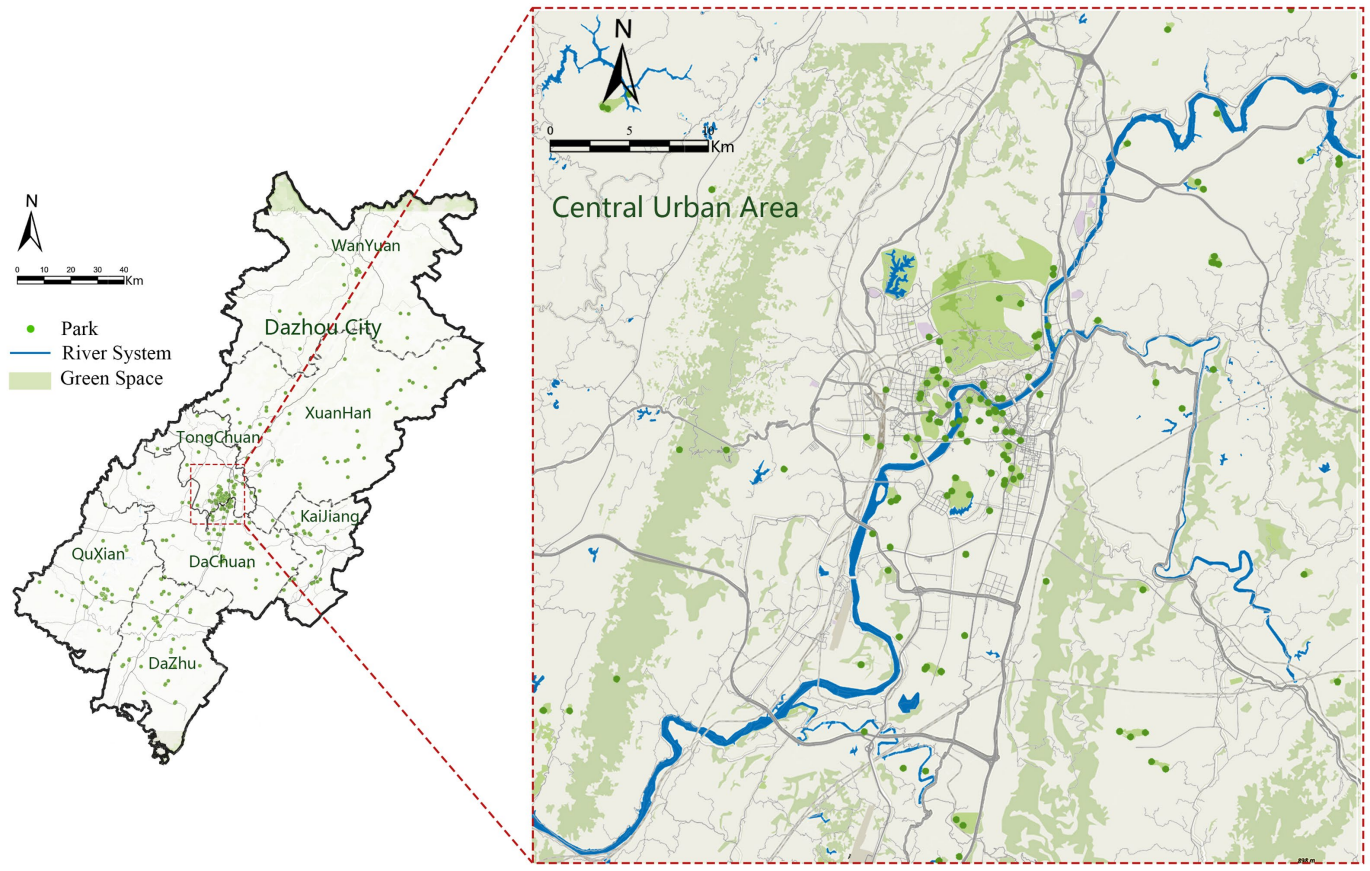
(Study area), the base map is sourced from the Guihuayun Website (https://guihuayun.com/maps/index.php). On the basis of this original base map, the author have independently redrawn and compiled the figure for this study. Base maps from: Guihuayun Maps (https://guihuayun.com/maps/).

The study area is Dazhou City, Sichuan Province, China, a regional central city at the junction of Sichuan, Chongqing, and Shaanxi. By 2020, the green coverage rate of Dazhou’s urban area had reached 38%, and a per capita park green space area of 13.46 m^2^. The city has been successfully recognized as a Provincial Garden City, and its proactive child-friendly urban policies provide a solid practical foundation for this research. Based on the current status of Dazhou’s green space system, this study conducted a typological classification of parks within the urban area (Figure 1). The selected research objects included comprehensive parks, sports-themed parks, waterfront wetland parks, community parks, and pocket green spaces, all of which were equipped with children’s outdoor activity areas, playgrounds, and sports facilities. These parks comprehensively reflect children’s activity patterns across diverse spatial types, thereby providing a valid and consistent observation basis for analyzing the influence of park environments on children’s health, while ensuring both the validity of the research and the representativeness of the findings.

### 2.2 Research framework

**Figure 2:**
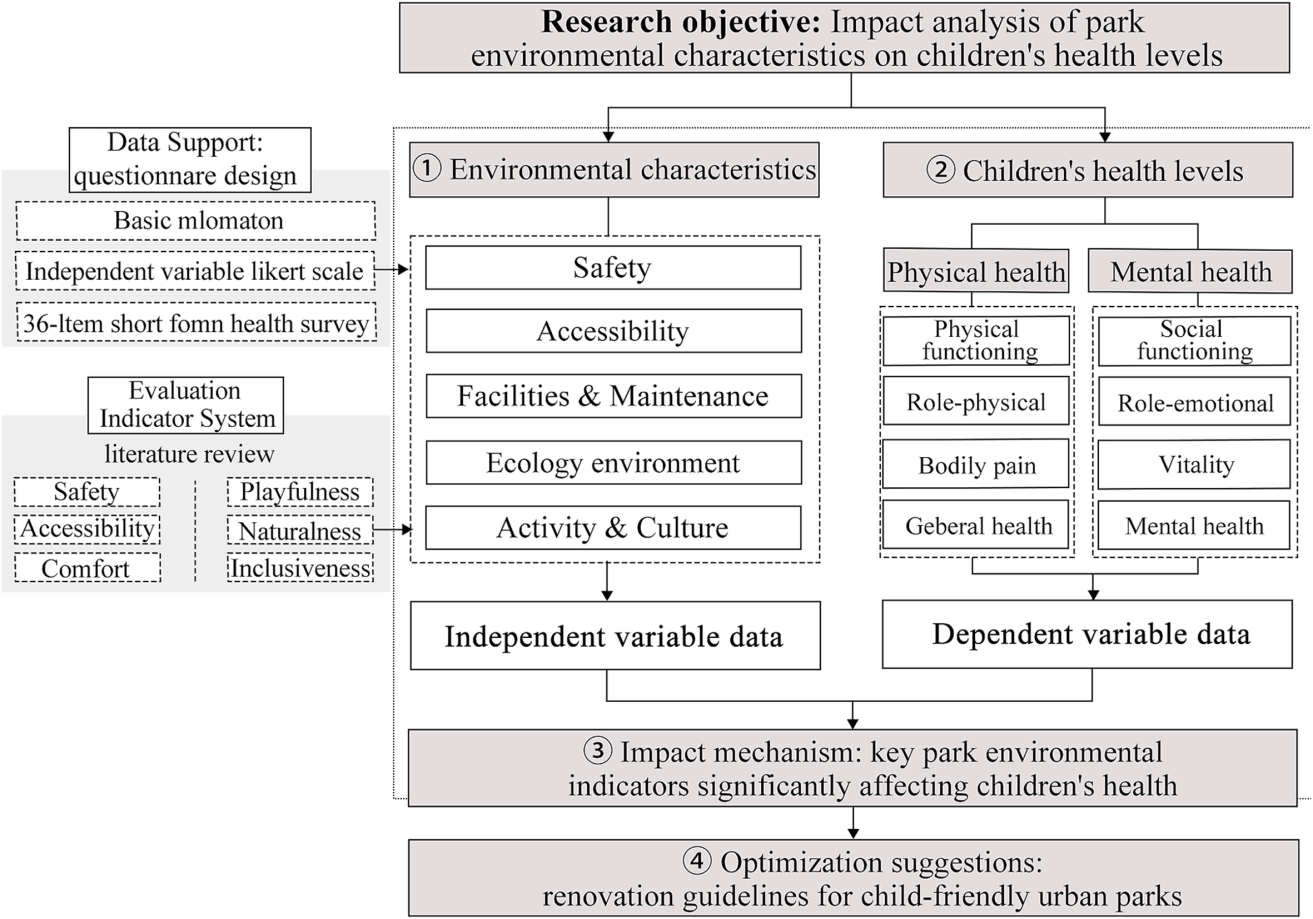
Research framework. The flowchart illustrates the stepwise analytical procedure, ranging from indicator construction and questionnaire distribution to factor analysis, correlation analysis, and multiple linear regression. It demonstrates how the study systematically assesses the impacts of park environmental characteristics on children’s physical and mental health.

This study collected data on urban park environmental characteristics in Dazhou City using a Likert-scale questionnaire and conducted a factor analysis to develop an evaluation index system encompassing six dimensions: safety, accessibility, comfort, playfulness, naturalness, and inclusiveness. The research framework integrates environmental characteristics, behavioral support, and health effects into a systematic assessment model, providing comprehensive and structured recommendations for the renewal and improvement of child health-oriented park design (see Figure 2).

### 2.3 Comprehensive evaluation framework for child-friendly spaces

Previous theoretical and literature reviews have confirmed six core dimensions: safety, accessibility, comfort, playfulness, naturalness, and inclusiveness. On this basis, this study further systematically reviewed relevant research outcomes, including the Delft Street Child-Friendliness Assessment System (42), evaluation indicators for improving child and adolescent friendliness (55), the Hierarchical Needs Model for Children (56), and key factors influencing children’s activities to extract spatial features that meet the health needs of children and adolescents.

Centering on the above six dimensions, further integrate and supplement the multiple collected indicators. For instance: In the safety category, safety requirements for accessible green spaces (57) and safety standards for eco-communities (58) were integrated, emphasizing monitoring facility coverage. In the naturalness category, design elements such as rain gardens and wildlife habitats were introduced to achieve the balance between ecology and applicability (58). In the playfulness category, drawing on research on activity site diversity (59) and spatial multi-functionality (60), indicators such as facility age appropriateness and terrain richness were included. In the accessibility and comfort categories, supplementary indicators were added, such as convenience of park entrances, pedestrian network connectivity, noise control, and seating density (54, 61).

Ultimately, this study condensed and formed a comprehensive evaluation framework comprising 6 criterion layers and 40 specific indicators (Table 1), laying a foundation for the subsequent analysis of the relationship between park environmental characteristics and child health outcomes.

**Table 1:** Health-oriented child-friendly spatial design indicators.

Primary indicator	Secondary indicators and single items	
Safety	Traffic safety	Pedestrian system isolation, vehicle speed management, crosswalk safety
	Public security	Monitoring facility coverage, nighttime lighting intensity, emergency call devices
	Facility safety	Maintenance of play equipment, protective edges, anti-slip materials
Accessibility	Spatial accessibility	Convenience of park entrances, pedestrian network connectivity
	Service accessibility	Distance to public service facilities
	Natural accessibility	Green space permeability, greenway connectivity
Comfort	Physical comfort	Pavement smoothness, shading coverage, seating density
	Environmental comfort	Noise control, air quality, sanitation and cleanliness
	Mental comfort	Sky visibility, parental supervision visibility
Playfulness	Play facility	Age-appropriate classification, facility diversity
	Spatial experience	Terrain richness, openness of play space, natural exploration zones
	Interactive design	Interactive engagement, educational/scientific features
Naturalness	Vegetation quality	Plant diversity, tree canopy coverage, seasonal landscape layering
	Ecological function	Rain garden design, wildlife habitat presence
Inclusiveness	Spatial flexibility	Multi-functional activity spaces, variable terrain features
	Social inclusiveness	Barrier-free design, multi-age shared spaces
	Cultural vitality	Local cultural elements, capacity for hosting festivals and events

### 2.4 Data sources

#### 2.4.1 Questionnaire design

The questionnaire was divided into three sections: basic information, park environmental characteristics (independent variables), and children’s physical and mental health (dependent variables). A mixed-scale design was employed to balance the objective data collection and subjective perception evaluation. Unlike studies relying on objective spatial attributes such as density, distance, or land-use mix, subjective evaluation can directly capture children’s actual perceptual experiences, including mental dimensions such as satisfaction, sense of belonging, and sense of security—key mediating pathways through which environmental characteristics affect health outcomes. Although objective measurement systems such as the “5D” framework (density, diversity, design, destination accessibility, and distance to transit) have been widely applied in research on adult travel behavior and public health, their applicability to children is limited. Children’s activity patterns, cognitive abilities, and environmental perception often differ from objective spatial indicators. For example, children’s perception of “accessibility” may be more strongly shaped by safety, playfulness, or accompaniment conditions rather than actual physical distance. Therefore, subjective evaluation is more suitable for reflecting children’s real usage experience and its potential impacts on health.

The basic information section collected demographic data including children’s age, sex, height, weight, as well as behavioral data covering daily outdoor activity duration, time period, and activity type. The park environmental characteristics section-representing independent variables which covered the six primary dimensions and forty single indicators described above, with a 5-point Likert scale used to evaluate both satisfaction and importance for each indicator, supporting factor variance analysis and Importance-Performance Analysis (IPA). The children’s physical and mental health section adopted the 36-Item Short Form Health Survey (62). Although originally designed for adults, it has been adapted for school-aged children and adolescents, demonstrating good reliability and validity in self-assessment of physical and mental health (63). Thus, the questionnaire design ensured the control of demographic and behavioral characteristics while empirically testing the hypothesized environment–health relationships using dual environmental and health measurement scales. The full questionnaire is provided in Supplementary Table 1.

#### 2.4.2 Data collection

To ensure authenticity and reliability, questionnaires were distributed in children’s outdoor activity areas, playgrounds, and sports facilities within the selected parks. The survey participants were children under 18 years of age. Field surveys were conducted in December 2024. Considering that some respondents might have difficulty completing the questionnaire independently, the survey adopted a semi-structured interview–assisted approach. Specifically, for children under 6 years of age, health-related information was collected by researchers through interviews with parents or guardians; for children aged 6 years and above, the questionnaire was completed with researcher assistance. A total of 236 questionnaires were distributed, of which 19 were invalid due to incomplete data. Hence, 217 valid responses were obtained, yielding an effective response rate of 92%.

## 3 Results

### 3.1 Evaluation system of child health-oriented urban park environmental characteristics

#### 3.1.1 Reliability and validity analysis

To ensure the reliability and validity of the questionnaire, a pretest was conducted before the formal survey. A total of 30 pretest questionnaires were distributed to evaluate the clarity of wording, the logical sequence of items, and the response time. Based on feedback from respondents, the wording of some items was revised and optimized. Reliability analysis was performed using Cronbach’s *α* coefficient to examine the internal consistency of the questionnaire. The overall Cronbach’s α coefficient of the questionnaire was 0.839, which is above the threshold of 0.70, indicating that the questionnaire had good internal consistency.

Before conducting factor analysis, it was necessary to determine the applicability of the data through a validity test, mainly by referencing the Kaiser-Meyer-Olkin (KMO) measure and Bartlett’s Test of Sphericity. The test results (Table 2) show that the KMO value was 0.812, exceeding the ideal threshold of 0.6, which indicates a satisfactory degree of overlap among the factors. Meanwhile, Bartlett’s Test of Sphericity yielded a chi-square statistic of 2708.554 (degrees of freedom = 780) with a significance level of *p* = 0.000 < 0.001, demonstrating that the survey data obtained in this study had high validity and that the factors were correlated.

**Table 2:** Results of KMO and Bartlett’s Test of Sphericity.

KMO measure of sampling adequacy	Bartlett’s Test of Sphericity		
	Approx. Chi-Square	df	Sig.
0.812	2708.554	780	0.000

“df”: reflect the quantity of independent information in data. “sig”: If sig < 0.05, the data is suitable for factor analysis; if sig ≥ 0.05, the data is not suitable for factor analysis.

#### 3.1.2 Establishment of the evaluation index system

The study adopted factor analysis using SPSS software, extracting principal components with eigenvalues greater than one as common factors. A total of 12 principal components were extracted, comprising 40 indicators, suggesting that these variables effectively represent information related to children’s health in park spaces. Due to overlapping items and low variance contribution rates among certain indicators, 10 items were removed (Appendix). Subsequently, a secondary classification of indicators was conducted, and the weights of the criterion and indicator layers were determined by calculating the variance contribution rates for each factor. Finally, a child health-oriented evaluation index system for urban park environmental characteristics was established (Table 3). The weight analysis results revealed variations in the relative importance of the five criterion layers for children’s health. Among them, the ecology environment dimension had the highest weight (0.311), highlighting the central role of natural exposure and environmental quality in promoting children’s health. The safety dimension ranked second (0.238), emphasizing that safety assurance is a fundamental prerequisite for children’s outdoor activities. The activity and cultural dimension (0.201) indicated that spatial design capable of stimulating social interaction, play behavior, and cultural identity is also indispensable. In comparison, the accessibility (0.136) and facility maintenance (0.114) dimensions had lower weights, but as supportive elements, they directly influenced the experiential quality of parks.

**Table 3:** Child health-oriented evaluation system for urban park environmental characteristics.

Criterion layer	Weight	Indicator layer	Indicator code	Weight
Ecology environment	0.311	Plant diversity	A1	0.748
		Nature exploration zone	A2	0.748
		Tree canopy coverage	A3	0.690
		Shade coverage	A4	0.606
		Air quality	A5	0.739
		Seasonal landscape	A6	0.607
		Green space permeability	A7	0.679
		Terrain richness	A8	0.496
		Wildlife habitat	A9	0.463
Safety	0.238	Barrier-free design	B1	0.716
		Vehicle speed management	B2	0.760
		Noise control	B3	0.573
		Anti-slip material	B4	0.711
		Night lighting intensity	B5	0.721
		Monitoring facility coverage	B6	0.497
		Parent supervision visibility	B7	0.449
Activity & culture	0.201	Multi-age shared zone	C1	0.776
		Age-appropriate zone	C2	0.697
		Interactive engagement	C3	0.520
		Local cultural element	C4	0.531
		Multi-functional activity zone	C5	0.696
		Educational facility	C6	0.522
Accessibility	0.136	Pedestrian system isolation	D1	0.735
		Pedestrian system connectivity	D2	0.670
		Park entrance accessibility	D3	0.576
		Pavement smoothness	D4	0.541
Facilities & maintenance	0.114	Facility diversity	E1	0.569
		Seating distribution density	E2	0.489
		Sanitation and cleanliness	E3	0.529
		Playground facility maintenance	E4	0.522

Weights reflect the relative contribution of each indicator to the overall evaluation framework. Higher weights indicate greater importance in explaining children’s health outcomes.

### 3.2 The impact of park environments on children’s health

#### 3.2.1 Descriptive statistics

A one-way analysis of variance (ANOVA) was employed to examine the differences in children’s and adolescents’ health outcomes under different covariates. Statistical significance was set at *p* < 0.05. The results show that for all three groups, the *p*-values confirmed that there were no significant differences in either physical or mental health scores among the subgroups under these covariates. Therefore, the mean values and standard deviations of each comparison group were used for subsequent descriptive interpretation.

Regarding age structure, both physical and mental health evaluations among children and adolescents were lowest in the “11–15 years” subgroup, while the sample size was largest in the “6–10 years” subgroup. In terms of duration of activity, physical health scores reached their highest mean value in the “2–3 h” subgroup, whereas mental health scores peaked in the “1–2 h” group. This suggests that moderate playtime should be encouraged, and that park spaces should include areas of sufficient size to attract children and adolescents to stay for extended period. With respect to time-of-day distribution, the “10-12 a.m.” subgroup demonstrated higher physical health scores with relatively stable variation (M = 72.63, SD = 6.89), indicating that this period may correspond to optimal levels of physical activity among children and adolescents. Meanwhile, the “14–16 p.m.” subgroup exhibited higher mental health scores (M = 50.95, SD = 6.29), suggesting that afternoon hours may be more conducive to mental relaxation and social interaction.

Based on the analyses of age structure, activity duration, and time-period distribution, the following integrated recommendations are proposed: (1) Focus on the 6–15 age group, designing health-promoting activities aligned with their developmental needs, such as team sports and creative games; (2) Plan outdoor activities or exercise programs during “10-12 a.m.” and “14–16 p.m.,” to leverage these periods’ potential physical and mental advantages; (3) Expand multifunctional zones within parks, such as sports areas, social corners, and nature-exploration zones, to attract children and adolescents to autonomously engage in suitable activities at different times of day, prolonging park stay duration and enhancing overall health benefits (see Table 4).

**Table 4:** Variance analysis of covariates and child health.

Dependent variable	Covariate		Sample size	Average	Standard deviation	F	P
Physical health	Age structure	Under the age of 5	57	71.82	9.26	0.524	0.666
		6–10 years	126	72.75	7.44		
		11–15 years	29	70.76	8.55		
		16-20 years	5	71.80	12.87		
		Total	217	72.22	8.20		
Mental health		Under the age of 5	57	49.05	7.91	1.943	0.124
		6–10 years	126	51.22	6.33		
		11–15 years	29	48.79	5.95		
		16-20 years	5	49.60	6.03		
		Total	217	50.29	6.78		
Physical health	Activity length	within 1 h	60	71.35	8.43	0.882	0.451
		1–2 h	115	72.69	8.80		
		2–3 h	32	73.06	5.16		
		more than 3 h	10	69.30	7.57		
		Total	217	72.22	8.20		
Mental health		within 1 h	60	49.93	6.87	0.833	0.477
		1–2 h	115	50.81	7.17		
		2–3 h	32	49.94	4.70		
		more than 3 h	10	47.60	7.20		
		Total	217	50.29	6.78		
Physical health	Time period distribution	10-12 a.m.	40	72.63	6.89	0.671	0.571
		12-14 p.m.	8	68.25	8.81		
		14–16 p.m.	44	72.50	9.02		
		16-18 p.m.	125	72.24	8.28		
		Total	217	72.22	8.20		
Mental health		10-12 a.m.	40	50.05	5.60	0.296	0.828
		12-14 p.m.	8	48.75	7.39		
		14–16 p.m.	44	50.95	6.29		
		16-18 p.m.	125	50.23	7.28		
		Total	217	50.29	6.78		

"F": The larger the F-value, the more obvious the difference between groups; the closer the F-value is to 1, the smaller the difference between groups. "P": If *p* < 0.05, the null hypothesis is rejected, and it is considered that there is a statistically significant difference between groups. If *p* ≥ 0.05, the null hypothesis cannot be rejected, and the result has no statistical significance.

#### 3.2.2 Correlation test

**Figure 3:**
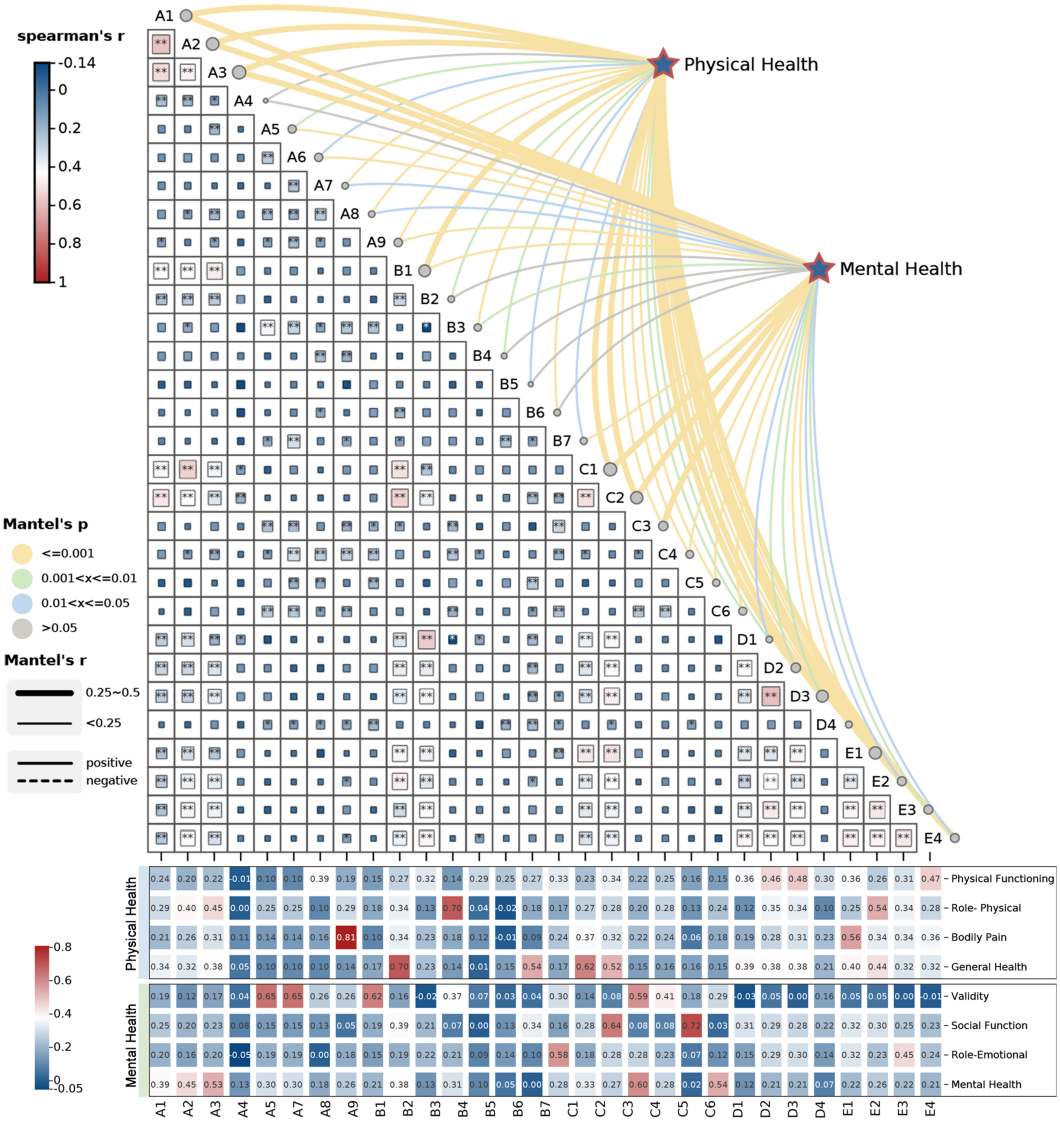
Correlation matrix between park environmental characteristics and children’s health.

To further investigate the intrinsic relationships between various park environmental characteristics and children’s health levels, this study employed the Mantel test to assess the correlations between environmental indicators and both physical and mental health of children. The results revealed that most environmental indicators exhibited significant correlations with children’s physical and mental health, although the strength and scope of the influence varied across indicators (Figure 3). It is noteworthy that shade coverage (Mantel’s r < 0.25, *p* > 0.05) was the only indicator without a significant correlation, while all other indicators demonstrated varying degrees of association with components of physical health, such as physical functioning, role-physical, bodily pain, and general health. At the mental health level, the Mantel test identified seven strongly correlated indicators (Mantel’s r = 0.25–0.5, *p* < =0.01), with a more concentrated composition. These include multi-age shared zone, plant diversity, natural exploration zone, barrier-free design, age-appropriate zone, tree canopy coverage, and interactive engagement.

In contrast, four indicators including shade coverage, anti-slip material, night lighting intensity, and monitoring facility coverage showed no significant correlation with mental health (Mantel’s r < 0.25, *p* > 0.05). Other environmental characteristics were found to be moderately correlated with components of mental health, such as vitality, social functioning, role–emotional, and mental health. In summary, multi-age shared zone, plant diversity, natural exploration zone, barrier-free design, age-appropriate zone, and tree canopy coverage were strongly correlated with both children’s physical and mental health, identifying them as core environmental elements for achieving comprehensive child health development. Interactive engagement was strongly correlated only with mental health, emphasizing its unique value in emotional satisfaction.

Conversely, indicators such as facility diversity and seating distribution density were primarily correlated with physical health, reflecting their supportive roles in facilitating physical activity. Safety-related factors such as anti-slip material, Night lighting intensity and monitoring facility coverage exhibited weaker associations with mental health, suggesting that they function more as implicit safeguards-essential prerequisites for activity-rather than as direct regulators of emotional well-being. Based on the above correlation results, and to ensure precision and parsimony in subsequent multiple linear regression models, this study made the following adjustments: in the physical health model, the indicator shade coverage was excluded; in the mental health model, four indicators including shade coverage, anti-slip material, night lighting intensity, and monitoring facility coverage were excluded.

#### 3.2.3 Collinearity diagnostics

Collinearity diagnostics in SPSS were used to further assess the severity of multicollinearity. The variance inflation factor (VIF) is commonly adopted for this purpose; a VIF value greater than 5 is generally considered indicative of multicollinearity. As shown in Table 5, the VIF values of the indicators in this study ranged from 1 to 3, further confirming the absence of collinearity.

**Table 5:** Collinearity diagnostics.

N	Indicator	VIF
A1	Plant diversity	2.221
A2	Nature exploration zone	2.566
A3	Tree canopy coverage	2.182
A4	Shade coverage	1.245
A5	Air quality	1.588
A6	Seasonal landscape	1.530
A7	Green space permeability	1.398
A8	Terrain richness	1.456
A9	Wildlife habitat	1.349
B1	Barrier-free design	2.150
B2	Vehicle speed management	1.891
B3	Noise control	1.607
B4	Anti-slip material	1.268
B5	Night lighting intensity	1.260
B6	Monitoring facility coverage	1.390
B7	Parent supervision visibility	1.464
C1	Multi-age shared zone	2.369
C2	Age-appropriate zone	2.381
C3	Interactive engagement	1.358
C4	Local cultural element	1.353
C5	Multi-functional activity zone	1.281
C6	Educational facility	1.394
D1	Pedestrian system isolation	2.22
D2	Pedestrian system connectivity	2.192
D3	Park entrance accessibility	2.184
D4	Pavement smoothness	1.319
E1	Facility diversity	2.115
E2	Seating distribution density	1.89
E3	Sanitation and cleanliness	2.158
E4	Playground facility maintenance	1.801

VIF values below 5 indicate that multicollinearity is not a concern, allowing stable estimation of regression coefficients.

#### 3.2.4 Multiple linear regression analysis

**Figure 4:**
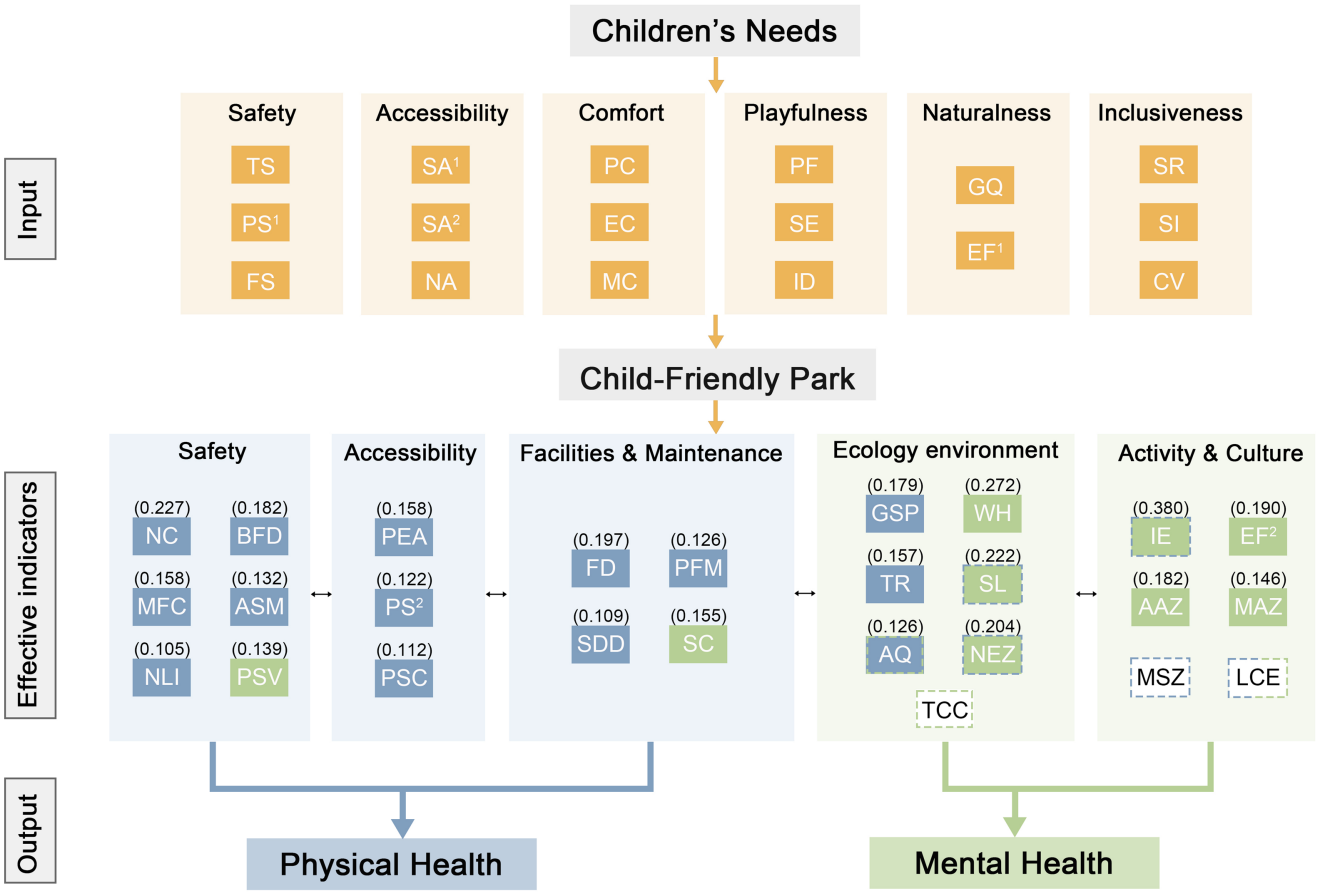
Mechanism of the impact of park environmental characteristics on children’s health. Keys of abbreviated terms: TS (Traffic Safety), PS1 (Public Safety), FS (Facility Safety), SA1 (Spatial Accessibility), SA2 (Service Accessibility), NA (Natural Accessibility), PC (Physical Comfort), EC (Environmental Comfort), MC (Mental Comfort), PF (Play Facility), SE (Spatial Experience), ID (Interactive Design), GQ (Greening Quality), EF1 (Ecological Function), SR (Spatial Resilience), SI (Social Inclusion), CV (Cultural Vitality), NC (Noise Control), MFC (Monitoring Facility Coverage), NLI (Night Lighting Intensity), BFD (Barrier-Free Design), ASM (Anti-Slip Material), PSV (Parent Supervision Visibility), PEA (Park Entrance Accessibility), PS2 (Pavement Smoothness), PSC (Pedestrian System Connectivity), FD (Facility Diversity), SDD (Seating Distribution Density), PFM (Playground Facility Maintenance), SC (Sanitation and Cleanliness), GSP (Green Space Permeability), TR (Terrain Richness), AQ (Air Quality), WH (Wildlife Habitat), SL (Seasonal Landscape), NEZ (Nature Exploration Zone), TCC (Tree Canopy Coverage), IE (Interactive Engagement), AAZ (Age-Appropriate Zone), MSZ (Multi-Age Shared Zone), EF2 (Educational Facility), MAZ (Multi-Functional Activity Zone), LCE (Local Cultural Element). A solid blue/green box indicates that the indicator is significantly associated with children’s physical/mental health, and the number in the box represents the significance coefficient. A dashed blue/green box indicates that the indicator has a certain linear association with children’s physical/mental health. The dashed box for LCE has two colors, indicating that LCE has a certain linear association with both children’s physical and mental health.

To accurately quantify the degree to which various environmental characteristics influence children’s health and identify their core driving factors, multiple linear regression analysis was conducted. The results revealed 14 indicators that exhibited significant linear relationships with children’s physical health, and 9 indicators that showed significant linear relationships with children’s mental health. The partial regression coefficients of these 23 indicators were further analyzed, systematically revealing the mechanisms through which environmental characteristics affect children’s health (Figure 4).

In the regression model for children’s physical health level, the model R^2^ = 0.893 > 0.5 (*p* < 0.001), indicating that the selected environmental variables effectively explained nearly 90% of the variance in children’s physical health. The regression results of the 14 indicators, ranked by standardized regression coefficient (*β*), are as follows: noise control (β = 0.227), facility diversity (β = 0.197), barrier-free design (β = 0.182), green space permeability (β = 0.179), terrain richness (β = 0.157), park entrance accessibility (β = 0.153), monitoring facility coverage (β = 0.153), anti-slip material (β = 0.132), playground facility maintenance (β = 0.126), air quality (β = 0.126), pavement smoothness (β = 0.122), pedestrian system connectivity (β = 0.112), seating distribution density (β = 0.109), and night lighting intensity (β = 0.105). These β values represent the expected change in physical health scores for a one-standard-deviation increase in each predictor, holding other variables constant. For example, a one-standard-deviation increase in noise control was associated with a 0.227 standard deviation increase in physical health scores, representing a moderate-to-strong effect. Together with air quality, it helps reduce the physical stress load imposed by environmental stimuli on children’s immune and cardiovascular systems. Facility diversity directly promotes moderate- to high-intensity physical activity by offering a wide range of play and exercise options. Barrier-free design, anti-slip material, monitoring facility coverage, and night lighting intensity jointly lower physical barriers and perceived risks, thus ensuring safety and encouraging participation in physical activity. Naturalness and interest-related indicators, such as green space permeability and terrain richness, stimulate spontaneous and exploratory physical engagement through mechanisms of “nature empowerment” thereby amplifying physical health benefits.

In the regression model for children’s mental health level, the model R^2^ = 0.870 > 0.5 (*p* < 0.001). The regression results of the 9 indicators, ranked by standardized regression coefficient (*β*), are as follows: interactive engagement (β = 0.380), wildlife habitat (β = 0.272), seasonal landscape (β = 0.222), natural exploration zone (β = 0.204), educational facility (β = 0.190), age-appropriate zone (β = 0.182), sanitation and cleanliness (β = 0.155), multi-functional activity zone (β = 0.146), and parental supervision visibility (β = 0.139). The results demonstrate that interactive engagement is the core factor, with an influence far exceeding that of other indicators. Through its synergy with age-appropriate zone, multi-functional activity zone, and educational facility, it fulfills children’s intrinsic needs for play, exploration, and social interaction, thereby fostering a sense of achievement and belonging, which serves as the core foundation for developing positive emotions and mental resilience. Furthermore, wildlife habitat, seasonal landscape, and natural exploration zone form another powerful pathway by providing “softly fascinating” natural stimuli, these elements stimulate curiosity and empathy, effectively enhancing attention restoration and reducing mental stress. Notably, parental supervision visibility, as part of the safety monitoring dimension, also showed a significant positive impact on mental health. This finding confirms that a perceived sense of safety is a crucial precondition for children to relax, engage deeply in play, and achieve mental restoration.

## 4 Discussion

Using empirical data from Dazhou City, this study verified the differentiated impact pathways through which the environmental characteristics of urban parks influence children’ s physical and mental health, and this finding transcends the generalized assumption that “green spaces are inherently beneficial to health” and reveals that different environmental attributes operate via distinct physical and mental mechanisms. In Dazhou’s urban parks, the promotion of children’s physical health primarily relies on a “safety and vitality–based environmental foundation”. Noise control, facility diversity, and barrier-free design jointly constitute a low-risk, low-stress, and physically stimulating spatial framework. This finding is highly consistent with SRT, which posits that negative environmental stimuli activate the hypothalamic–pituitary–adrenal and trigger stress responses. In contrast, low-disturbance and high-controllability environments can reduce cortisol levels and sympathetic nerve activity, thereby improving immune function and alleviating cardiovascular burden (61). Thus, effective noise control is a prerequisite for mental recovery and stability. Facility diversity, by providing varied opportunities for climbing, running, and ball games, directly promotes moderate- to high-intensity physical activity, which is crucial for preventing obesity, bone development, and cardiopulmonary improvement (64). The prominent role of barrier-free design underscores that it not only serves children with special needs but also reduces physical risks and fatigue for all children and encourages autonomous exploratory behavior by creating smooth, safe, and accessible spaces.

In contrast, the promotion of children’s mental health depends on mental satisfaction and restorative experiences, a process that can be explained by ART. This theory emphasizes that the “soft fascination” in natural environments helps restore attention, reduce mental fatigue, and provide pathways for emotional engagement, thereby eliciting positive emotions. Its core driving forces differ substantially from those of physical health. Highly interactive play facilities and biophilic installations satisfy children’s intrinsic needs for play and social connection, fostering a sense of achievement and belonging, which forms the core mechanism for cultivating positive emotions and mental resilience (65). Wildlife habitat and rich seasonal landscape provide children with opportunities to connect with natural life processes. Such “soft fascination” elements stimulate curiosity and empathy, effectively reducing stress, restoring attention. This also explains why naturalness and interactivity exert stronger effects on mental health than purely safety-oriented characteristics. Even physically safe parks may yield limited mental benefits if they lack opportunities for participation, exploration, and connection with living systems. Therefore, urban parks in Dazhou especially comprehensive parks and waterfront wetland parks-should transition from a focus on “aesthetic landscaping” to “experiential creation” This can be achieved by incorporating interactive installations, preserving natural habitat, and designing plant communities rich in seasonal dynamics, thereby meeting children’s mental needs (66).

Importantly, this study identified several previously overlooked but highly influential environmental characteristics. For instance, the significant effects of green space permeability and terrain richness on physical health indicate that in high-density urban contexts with limited land resources, it is unnecessary to rely solely on large, centralized green spaces. Instead, Micro-scale green infill strategies can increase the frequency of interaction between humans and nature. Fragmented and embedded natural elements encourage children’s independent exploratory behavior and extend the duration of outdoor activities. These findings challenge the traditional emphasis on large-scale centralized green spaces and support a more flexible green infill theory (67).

Furthermore, the significant impact of parental supervision on children’s mental health highlights a social-environmental mediation mechanism shaped by the Chinese cultural context. Thus, “caregiver-friendliness” should become an indispensable component of child-friendly park design in Dazhou and other Chinese cities in the future. Optimizing seating layouts and eliminating visual blind spots can reduce parental anxiety, thereby indirectly creating a freer and more encouraging atmosphere for children’s activities to take place. Unlike some western studies that emphasize the role of shading in mitigating heat stress (68), this study found that shade coverage had no significant impact. This may be attributed to local behavioral norms, as most outdoor activities of children in Dazhou occur during mild morning and evening hours, reducing the immediate need for shade. Moreover, given the generally high vegetation coverage within the humid subtropical climate of the Sichuan Basin, it may be more effective to focus on improving the site ventilation design. Similarly, the insignificant relationship between night lighting and mental health aligns with daytime-oriented activity patterns observed in the sample. These results suggest that environmental effects are not universal, but are contingent on and moderated by behavioral practices, climatic conditions, and cultural norms.

More importantly, this study identified a set of key design indicators, including barrier-free design, parental supervision visibility, and pavement smoothness, that have received limited attention in Western literature yet are critical in high-density urban settings and within China’s intergenerational caregiving framework. These factors highlight the urgent need for spatial refinement, safety assurance, and culturally specific childcare practices in cities like Dazhou, providing new, actionable intervention targets for optimizing child-friendly park environments in China. Although local cultural elements did not reach statistical significance, their potential linear correlation underscores the unique value of cultural integration. This implies that incorporating cultural symbols into age-appropriate retrofitting of urban environments may indirectly strengthen children’s sense of environmental belonging and cultural identity, thereby promoting social equity and governance legitimacy.

## 5 Conclusion

Based on empirical research conducted in Dazhou City, a high-density urban area in China, this study systematically analyzed the mechanisms through which urban park environmental characteristics influence children’s physical and mental health. By constructing and validating an evidence-based environmental indicator assessment system, this study further elucidates the differentiated impact pathways through which diverse environmental dimensions on children’s health outcomes, complementing the child-friendly city research framework that has long been dominated by Western theories.

The findings confirm that children’s physical health primarily depends on the establishment of a “safety and vitality foundation”. Among these, noise control, facility diversity, and barrier-free design play significant roles in ensuring safety and promoting physical activities. In contrast, children’s mental health relies more heavily on mechanisms of “mental satisfaction and restorative experience”, driven mainly by interactive engagement, wildlife habitat, and seasonal landscape that integrate natural and social interactions as core influences. Furthermore, this study identified several localized key indicators, such as terrain richness and parental supervision visibility, which are often overlooked in conventional design frameworks but demonstrate significant potential for health promotion within high-density urban contexts, providing new empirical evidence for the refined design of child-friendly parks in high-density cities.

Although this study has achieved certain advancements in both theoretical and empirical aspects, it still has several limitations that necessitate further prudent consideration. First, this study draws on a single-city sample. While Dazhou demonstrates a certain degree of representativeness regarding its climatic conditions, spatial structure and family care models, it also carries inherent regional specificities. Second, health data primarily relies on subjective self-reported data based on questionnaires (the SF-36 scale). Subjective data is usually influenced by factors such as mood, motivation, and expectations; thus, the research conclusions are more suitable for explaining the impact of the environment on “health perception and mental experience” rather than directly representing changes in physical health. Additionally, subjective data may be affected by emotional states, cognitive biases, and common method bias. Thus, future studies should incorporate objective biological markers, such as cortisol levels and heart rate variability, for longitudinal validation. Third, the research adopted a cross-sectional design, which limits the ability to assess the long-term health benefits of park optimization. Future research should extend across diverse geographic and cultural settings and integrate GIS-based spatial data, wearable monitoring devices, and other objective measurement technologies to conduct longitudinal studies. Such efforts would further elucidate the dynamic causal pathways among space, behavior, and health, providing a more robust scientific foundation for innovative paradigms in designing children’s activity spaces in high-density urban environments.

## Data Availability

The original contributions presented in the study are included in the article/Supplementary material, further inquiries can be directed to the corresponding author.

## Appendix

**Table A1:** Analysis of indicator weighting.

First-level indicator	Second-level indicator	Loading factor	Eigenvalue	Variance contribution rate/%
X1	Multi-age shared space	0.776	7.293	12.743
	Plant diversity	0.748		
	Natural exploration area	0.748		
	Universal design	0.716		
	Age-appropriate classification	0.697		
	Arbor coverage rate	0.690		
	Shade coverage rate	0.606		
	Facility diversity	0.569		
	Seat distribution density	0.489		
X2	Motor vehicle speed limit control	0.760	3.868	8.147
	Pedestrian system isolation	0.735		
	Pedestrian system connectivity	0.670		
	Park entrance convenience	0.576		
	Sanitation and cleanliness level	0.529		
	Amusement facility maintenance level	0.522		
X3	Air quality	0.739	2.241	6.556
	Seasonal landscape layers	0.607		
	Noise control	0.573		
	Facility science popularization value	0.522		
	Interactive participation	0.520		
X4	Material slip resistance	0.711	1.514	4.701
	Green space penetration rate	0.679		
	Local cultural element	0.531		
	Terrain richness	0.496		
X5	Multifunctional activity venue	0.696	1.449	4.294
	Slow-traffic pavement flatness	0.541		
	Biological habitat	0.463		
X6	Night lighting intensity	0.721	1.392	4.166
	Monitoring facility coverage rate	0.497		
	Parent supervision view	0.449		
