# Supplementary material for: Linking park environmental characteristics to child health outcomes: towards an evidence-based child-friendly design framework
Source: Front Public Health. 2026 May 15;14:1795835. doi: 10.3389/fpubh.2026.1795835 (PMC13220286; doi:10.3389/fpubh.2026.1795835)
Supplement: Supplementary file 1 [file Table_1.DOCX]

**Health-Oriented Satisfaction Rating Scale for Children and Adolescents' Parks in Dazhou City**

Survey Area Name: ________________ Questionnaire Number: ________

Dear Children/Respected Parents,

Hello! I am a postgraduate student from Northwest A&F University, conducting research on the topic "Study on Environmental Characteristics and Optimization Design of Children and Adolescents' Parks from the Perspective of Healthy Cities". This questionnaire will provide valuable data for this research, and I sincerely appreciate your assistance! The questionnaire will be filled out anonymously and used solely for academic research. Please feel free to complete it. Thank you!

a.What is the age of your child (hereinafter referred to as "the child")? ________

b.What is the child's gender? □ Male □ Female

c.The child's height: ________ cm; Weight: ________ kg

d.How long does the child usually play in outdoor spaces such as parks every day?

□ Less than 1 hour □ 1-2 hours □ 2-3 hours □ More than 3 hours

e.At which time period does the child go to play in outdoor spaces such as parks?

□ 7:00-10:00 □ 10:00-12:00 □ 12:00-14:00 □ 14:00-16:00 □ 16:00-18:00

**Table S1:Survey on Children's Needs for Park Environments**

|  |  | Evaluation Indicators | Satisfaction Rating | | | | | Importance Level | | | | |
| --- | --- | --- | --- | --- | --- | --- | --- | --- | --- | --- | --- | --- |
|  |  |  | Very Dissatisfied | Dissatisfied | Neutral | Satisfied | Very Satisfied | Very Unimportant | Unimportant | Neutral | Important | Very Important |
| Safety | Traffic Safety | Isolation of pedestrian system |  | | | | |  | | | | |
|  |  | Motor vehicle speed limit control |  | | | | |  | | | | |
|  |  | Safety of street-crossing facilities |  | | | | |  | | | | |
|  | Public Security Safety | Coverage rate of monitoring facilities |  | | | | |  | | | | |
|  |  | Night lighting intensity |  | | | | |  | | | | |
|  |  | Emergency call devices |  | | | | |  | | | | |
|  | Facility Safety | Maintenance degree of amusement facilities |  | | | | |  | | | | |
|  |  | Edge protection of facilities |  | | | | |  | | | | |
|  |  | Slip resistance of materials |  | | | | |  | | | | |
| Accessibility | Spatial Accessibility | Convenience of park entrances |  | | | | |  | | | | |
|  |  | Connectivity of pedestrian paths |  | | | | |  | | | | |
|  | Service Accessibility | Distance to public service facilities |  | | | | |  | | | | |
|  | Natural Accessibility | Penetration rate of green space |  | | | | |  | | | | |
|  |  | Connectivity of green slow-traffic paths |  | | | | |  | | | | |
| Comfort | Physical Comfort | Flatness of slow-traffic pavement |  | | | | |  | | | | |
|  |  | Shade coverage rate |  | | | | |  | | | | |
|  |  | Distribution density of seats |  | | | | |  | | | | |
|  | Environmental Comfort | Noise control |  | | | | |  | | | | |
|  |  | Air quality |  | | | | |  | | | | |
|  |  | Sanitation and cleanliness |  | | | | |  | | | | |
|  | Psychological Comfort | Sky visibility |  | | | | |  | | | | |
|  |  | Parents' supervision view |  | | | | |  | | | | |
| Interestingness | Game Facilities | Age-appropriate classification |  | | | | |  | | | | |
|  |  | Diversity of facilities |  | | | | |  | | | | |
|  | Spatial Experience | Terrain richness |  | | | | |  | | | | |
|  |  | Openness of game space |  | | | | |  | | | | |
|  |  | Natural exploration area |  | | | | |  | | | | |
|  | Interactive Design | Interactive participation |  | | | | |  | | | | |
|  |  | Popular science value of facilitie |  | | | | |  | | | | |
| Naturalness | Greening Quality | Plant diversity |  | | | | |  | | | | |
|  |  | Arbor coverage rate |  | | | | |  | | | | |
|  |  | Seasonal landscape layers |  | | | | |  | | | | |
|  | Ecological Function | Rain garden design |  | | | | |  | | | | |
|  |  | Biological habitats |  | | | | |  | | | | |
| Multifunctionality & Inclusion | Spatial Flexibility | Multifunctional activity venues |  | | | | |  | | | | |
|  |  | Terrain variability |  | | | | |  | | | | |
|  | Social Inclusion | Barrier-free design |  | | | | |  | | | | |
|  |  | Multi-age shared space |  | | | | |  | | | | |
|  | Cultural Vitality | Local cultural elements |  | | | | |  | | | | |
|  |  | Capacity to host festive activities |  | | | | |  | | | | |

**Table S2: Children's Physical and Mental Health (Dependent Variables)**

| **1.In general, how would you rate your health status/your child's health status?** ① Very good ② Good ③ Moderately good ④ Average ⑤ Poor | |
| --- | --- |
| **2.Compared with one year ago, how is your current health status?** ① Much better ② Slightly better ③ About the same ④ Slightly worse ⑤ Much worse  **3.The following questions are related to daily activities. Please think about whether your health status limits these activities, and to what extent?**  (1) Heavy physical activities (e.g., running, participating in intense sports):① Greatly limited ② Limited ③ Not limited at all  (2) Moderate activities (e.g., sweeping the floor, doing simple gymnastics):① Greatly limited ② Limited ③ Not limited at all  (3) Carrying daily necessities (e.g., buying groceries, shopping): ① Greatly limited ② Limited ③ Not limited at all  (4) Climbing several flights of stairs: ① Greatly limited ② Limited ③ Not limited at all  (5) Climbing one flight of stairs: ① Greatly limited ② Limited ③ Not limited at all  (6) Bending, kneeling, squatting: ① Greatly limited ② Limited ③ Not limited at all  (7) Walking a distance of more than 1500 meters: ① Greatly limited ② Limited ③ Not limited at all  (8) Walking a distance of 1000 meters: ① Greatly limited ② Limited ③ Not limited at all  (9) Walking a distance of 100 meters: ① Greatly limited ② Limited ③ Not limited at all  (10) Bathing and dressing independently: ① Greatly limited ② Limited ③ Not limited at all | |
| **4.In the past 4 weeks, have you encountered the following problems in your study and daily activities due to physical health issues?** | |
| (1) Reduced time spent on study or other activities: ① Yes ② No  (2) Only able to complete part of the things you wanted to do: ① Yes ② No  (3) Restricted types of study and activities you wanted to engage in: ① Yes ② No  (4) Increased restrictions on completing study or other activities: ① Yes ② No |  |
| **5.In the past 4 weeks, have you encountered the following problems in your work and daily activities due to emotional factors (depression and anxiety)?** | |
| (1) Reduced time spent on study or other activities: ① Yes ② No  (2) Only able to complete part of the things you wanted to do: ① Yes ② No  (3) Being less careful than usual when doing things: ① Yes ② No |  |
| **6.In the past 4 weeks, to what extent has your poor health or mood affected your social interactions with family, classmates, etc.?** | |
| ①No impact at all ② Slight impact ③ Moderate impact④ Significant impact ⑤ Very significant impact | |
| **7.In the past 4 weeks, have you experienced physical pain?** | |
| ① No pain at all ② Very slight pain ③ Slight pain ④ Moderate pain ⑤ Severe pain ⑥ Very severe pain | |
| **8.In the past 4 weeks, has physical pain affected your study and life?** | |
| ① No impact at all ② Slight impact ③ Moderate impact ④ Significant impact ⑤ Very significant impact | |
| **9.The following questions are about your feelings in the past month. Which answer best describes your situation for each question?** | |
| (1) You feel that life is fulfilling: ① All the time ② Most of the time ③ Quite often ④ Sometimes ⑤ Seldom ⑥ Never feel this way  (2) You are a sensitive person: ① All the time ② Most of the time ③ Quite often ④ Sometimes ⑤ Seldom ⑥ Never  (3) You are in a very bad mood and nothing can cheer you up: ① All the time ② Most of the time ③ Quite often ④ Sometimes ⑤ Seldom ⑥ Never  (4) You feel calm mentally: ① All the time ② Most of the time ③ Quite often ④ Sometimes ⑤ Seldom ⑥ Never  (5) You feel energetic when doing things: ① All the time ② Most of the time ③ Quite often ④ Sometimes ⑤ Seldom ⑥ Never  (6) You are in a low mood: ① All the time ② Most of the time ③ Quite often ④ Sometimes ⑤ Seldom ⑥ Never  (7) You feel exhausted: ① All the time ② Most of the time ③ Quite often ④ Sometimes ⑤ Seldom ⑥ Never  (8) You are a happy person: ① All the time ② Most of the time ③ Quite often ④ Sometimes ⑤ Seldom ⑥ Never  (9) You feel bored: ① All the time ② Most of the time ③ Quite often ④ Sometimes ⑤ Seldom ⑥ Never |  |
| **10.Has poor health affected your social activities (e.g., visiting relatives and friends)?**  ① All the time ② Most of the time ③ Quite often ④ Sometimes ⑤ Seldom ⑥ Never feel this way | |
| **11.Please look at each of the following questions and choose the answer that best matches your situation** | |
| (1) I seem to get sick more easily than others: ① Absolutely true ② Mostly true ③ Uncertain ④ Mostly false ⑤ Absolutely false  (2) I am as healthy as the people around me: ① Absolutely true ② Mostly true ③ Uncertain ④ Mostly false ⑤ Absolutely false  (3) I think my health status is deteriorating: ① Absolutely true ② Mostly true ③ Uncertain ④ Mostly false ⑤ Absolutely false  (4) My health status is very good: ① Absolutely true ② Mostly true ③ Uncertain ④ Mostly false ⑤ Absolutely false |  |

| Additional Suggestions: |
| --- |
